# Clinical predictors for a complicated course of disease in an inception cohort of patients with ulcerative colitis: results from the prospective, observational EPICOL study

**DOI:** 10.1007/s00384-022-04098-7

**Published:** 2022-01-27

**Authors:** Carsten Schmidt, Bernd Bokemeyer, Andreas Lügering, Dominik Bettenworth, Niels Teich, Imma Fischer, Leonie Hammer, Stefanie Kolterer, Stefan Rath, Andreas Stallmach, on behalf of the EPICOL Study Group

**Affiliations:** 1 Medical Clinic II, Fulda Hospital, Pacelliallee 4, Fulda, 36043 Germany; 2 Medical Faculty of the Friedrich Schiller University, Jena, Germany; 3 Interdisciplinary Crohn and Colitis Centre, Minden, Germany; 4 MVZ Portal 10, Münster, Germany; 5 Practice for Internal Medicine, Münster, Germany; 6 Practice for Internal Medicine, Leipzig, Germany; 7 Biostatistik-Tuebingen, Tübingen, Germany; 8 Medical Department, AbbVie Deutschland GmbH & Co. KG, Wiesbaden, Germany; 9 Clinic for Internal Medicine IV, Jena University Hospital, Jena, Germany

**Keywords:** Ulcerative colitis, Complicated disease, Predictors, Immunosuppressant, TNF-α antagonist, Biologics

## Abstract

**Purpose:**

The clinical course of ulcerative colitis (UC) is highly heterogeneous, with 20 to 30% of patients experiencing chronic disease activity requiring immunosuppressive or biologic therapies. The aim of this study was to identify predictors for a complicated disease course in an inception cohort of patients with UC.

**Methods:**

EPICOL was a prospective, observational, inception cohort (UC diagnosis, ≤ 6 months) study in 311 patients with UC who were naive to immunosuppressants (IS)/biologics. A complicated course of disease was defined as the need for IS and/or biologic treatment (here therapy with a TNF-α antagonist) and/or UC-related hospitalisation. Patients were followed up for 24 months.

**Results:**

Of the 307 out of 311 participants (4 patients did not meet the inclusion criteria “confirmed diagnosis of active UC within the last 6 months” (*n* = 2) and “immunosuppressive-naïve” (*n* = 2), analysis population), 209 (68.1%) versus 98 (31.9%) had an uncomplicated versus a complicated disease course, respectively. In a multivariate regression analysis, prior use of corticosteroids and prior anaemia were associated with a significantly increased risk for a complicated disease course (2.3- and 1.9-fold increase, respectively; *p* < 0.001 and *p* = 0.002). Based on these parameters, a risk model for patient stratification was developed.

**Conclusion:**

Our study identifies anaemia and an early need for corticosteroids as predictors for a complicated course of disease in an inception cohort of patients with UC. By determining these parameters in routine clinical practice, our results may support the identification of patients who might benefit from early escalation of therapy.

**Supplementary information:**

The online version contains supplementary material available at 10.1007/s00384-022-04098-7.

## Introduction

Ulcerative colitis (UC) is an inflammatory bowel disease (IBD) characterised by colonic inflammation extending proximally from the rectum [1]. Depending on disease localisation, UC may present as proctitis, left-sided colitis, or pancolitis [2]. Recurrent disease flares can lead to accumulating intestinal damage with long-term consequences, such as toxic megacolon and colectomy as well as an increased risk of colorectal cancer [3, 4].

Conventional management of UC is based on a step-up approach in which treatment is continually escalated if the therapeutic goals are not reached [5, 6]. Aminosalicylates, such as 5-aminosalicylic acid (5-ASA) with oral or rectal administration, are used as first-line medication, followed by oral or intravenous corticosteroid treatment if the disease remains uncontrolled. Patients with steroid dependency and frequent relapses can subsequently be treated with immunosuppressants (IS)/immunomodulators and advanced therapies, such as tumour necrosis factor–α (TNF-α) antagonists, anti-integrin antibodies, interleukin-12/23 antagonists, or Janus kinase inhibitors [5, 6].

Increasing evidence demonstrates the progressive nature of UC and thus emphasises the need for tight disease control that goes beyond the treatment of symptoms to avoid long-term accumulation of intestinal damage [7–9]. Updated treatment goals therefore comprise steroid-free remission as well as a normal quality of life and suggest objective indicators of disease activity, such as endoscopic remission [10]. Because achieving these therapeutic targets in patients with moderate to severe UC is often difficult, early initiation of IS or biologic treatment should be considered in patients with high risk for a complicated disease course [6, 11]. Therefore, early identification of patients who are at an increased risk for a complicated disease course is needed to be able to initiate appropriate therapy.

To enable effective identification of at-risk patients in daily clinical practice, easily assessable parameters for the prediction of individual disease courses are necessary. Numerous studies have already aimed to address this issue [12, 13]. Among others, these studies have identified young age at diagnosis, male sex, extensive colitis, severe endoscopic activity, and steroid dependency/resistance as predictors for hospitalisation, colectomy, and other complications [14–22]. As a result, risk stratification based on such parameters has been incorporated into various clinical decision guidelines [6, 23].

Despite this progress, many important questions and challenges remain. First, although the previously mentioned high-throughput techniques hold great promise, it will likely take many years before they can be established in routine clinical use, where cost and time are important limiting factors. Second, many studies on predictive parameters are retrospective and validation from prospective designs is frequently lacking. Finally, many investigated cohorts had a considerable disease duration or had already experienced some form of complications or therapy intensification. Predicting long-term outcomes based on prior events, such as hospitalisations, is certainly useful, but such complications should ideally be avoided in the first place by immediately identifying at-risk patients at diagnosis or shortly thereafter. Therefore, more studies in inception cohort patients are needed.

Here, we describe the results of EPICOL (Early Predictive Parameters of Immunosuppressive Therapy in Ulcerative Colitis). As with the forerunner study, EPIC, we aimed to prospectively validate numerous clinical predictors for a complicated disease course in patients recently diagnosed with UC, focusing in particular on parameters that are readily assessable in everyday clinical practice.

## Materials and methods

### Study design

EPICOL was a non-interventional, prospective, multicentre, observational study in 311 patients with UC as inception cohort (diagnosis, < 6 months) conducted between March 2015 and April 2020. All patients were naive to IS and biologics. Patients were enrolled consecutively at centres representing different levels of care, including outpatient hospital centres and gastroenterological practices in Germany. The endpoints were frequency of UC-related hospitalisation and therapy with IS or initiation of anti–TNF-α therapy. Six visits were scheduled: one at baseline and one each after 3, 6, 12, 18, and 24 months. Any unscheduled visit to a physician, outpatient visits to a hospital, or hospitalisation related to the UC diagnosis were documented as unscheduled visits. Patient demographics, such as age, sex, height, tobacco use, family members with UC, and medical history (e.g. date of first UC symptoms, date of UC diagnosis, previous and current use of non-immunosuppressive UC medication) were collected retrospectively for the status of diagnosis and documented at the baseline visit. In addition, disease symptoms and UC-related extraintestinal manifestations (EIMs), such as pyoderma gangrenosum, erythema nodosum, uveitis/iritis, arthralgia/arthritis, and ankylosing spondylitis, were assessed at every visit and collected retrospectively for the status of diagnosis. Weight and weight loss, localisation of disease, current surgical status, and UC medication or changes in medication (both IS/biologic and non-IS medication) were documented for every visit. Laboratory parameters (anaemia, platelets, faecal calprotectin, C-reactive protein, colonoscopy results, and disease activity according to the simple clinical colitis activity index (SCCAI)) were determined for every visit, if available. All above-mentioned parameters except patient demographics were assessed again at every visit after the baseline visit. The time interval until the need for IS and/or anti–TNF-α therapy and/or UC-related hospitalisation was documented.

EPICOL is a prospective study with the same design as the recently published EPIC (Early Predictive Parameters of Immunosuppressive Therapy in Crohn’s Disease) study, in which we investigated clinical parameters for their predictive power regarding the disease course in patients with recently diagnosed Crohn’s disease (CD) who were naive to treatment with both IS and biologics [24]. In that prospective cohort, we found that a complicated disease course, defined as the need for IS and/or anti–TNF-α treatment and/or CD-related hospitalisation, was significantly associated with various baseline criteria, namely, age at diagnosis < 40 years, anaemia, and treatment with systemic corticosteroids at first flare. Based on these parameters, a risk model in CD was developed that predicted a complicated disease course in our cohort with an accuracy of 87.2%.

### Patient population

In total, 311 patients with a confirmed diagnosis of active UC (except proctitis) no sooner than 6 months before the baseline visit who were ≥ 18 years of age, naive to treatment with conventional IS (thiopurines, calcineurin inhibitors, or equivalent therapy) and biologics (adalimumab, golimumab, infliximab, vedolizumab) at the baseline visit were enrolled consecutively. Pregnant women or patients with previous UC-related surgery were excluded from the study. Note that the use of tofacitinib and ustekinumab for UC was not approved in Europe until July 2018 and September 2019, respectively.

### Statistical analysis

Demographic and anamnestic data as well as case history and medication use were analysed descriptively. Mann–Whitney *U* tests, Fisher exact tests, and chi-square tests were conducted for comparison of the two groups (patients with vs without need for hospitalisation or immunosuppressive therapy).

To analyse predictive factors for the need of IS or hospitalisation within 24 months, uni- and multivariate Cox regression analyses were performed. Predictive factors considered overall were sex and clinical response to corticosteroid therapy; predictive factors at diagnosis were age and anaemia; at baseline visit were disease severity (SCCAI), Mayo endoscopic subscore of 3 (severe), and thrombocytosis; and at diagnosis or baseline visit were therapy with systemic corticosteroids, EIM, and smoking status. For the multivariate complete Cox regression model, the factors were examined with regard to co-linearity and interaction. Reduced Cox regression model was established with all factors, with *p* < 0.05 within univariate or multivariate complete Cox regression models. Parameters that were significant with *p* < 0.05 in this reduced model were then selected on a stepwise basis to obtain an optimised model. Based on the optimised model, a predictive risk model was developed to determine an individual patient’s probability of experiencing a complicated disease course at 6, 12, or 24 months.

## Results

### Patient characteristics and disease course

**Fig. 1:**
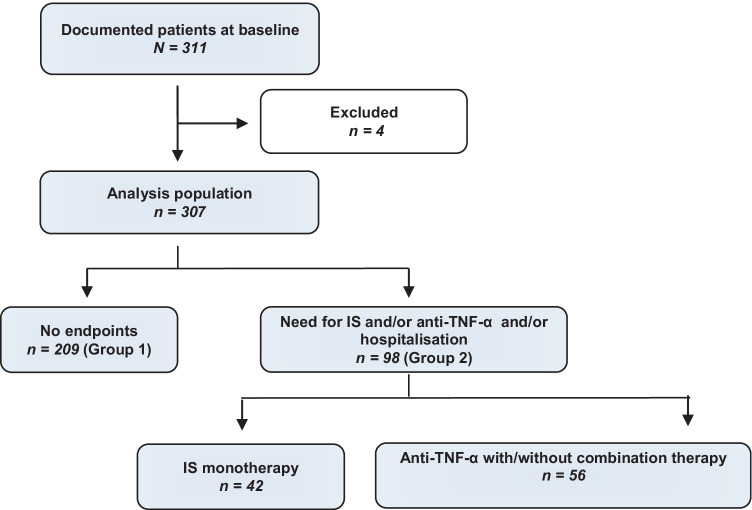
Patient disposition and analysis population. Reasons for exclusion (*n* = 4) were “confirmed diagnosis of active UC within the last 6 months” (*n* = 2) and “immunosuppressant-naive” (*n* = 2). IS, immunosuppressants; TNF-α, tumour necrosis factor–α

In total, an inception cohort of 311 patients with a recent diagnosis of UC (median time since diagnosis, 1.9 months) was enrolled in the study. Four patients who did not meet inclusion criteria were excluded (“confirmed diagnosis of active UC within the last 6 months” (*n* = 2) and “immunosuppressive-naïve” (*n* = 2)), and the final analysis population consisted of 307 patients with UC (Fig. 1). Baseline characteristics of the analysis population are shown in Table 1. The mean age was 38.5 years, and 44.6% (*n* = 137) were female.

**Table 1:** Patient characteristics at baseline

Parameter	Patients *n* = 307
Age, years	38.5 ± 15.6
Symptom duration, months	13.6 ± 32.6
Time since diagnosis, months	1.9 ± 1.9
BMI, kg/m^2^; *n* = 279	25.2 ± 5.2
Weight, kg; *n* = 280	77.2 ± 19.8
Current smoker, years; *n* = 43	15.5 ± 11.3
Time since stopped smoking, years; *n* = 66	6.6 ± 7.3
Faecal calprotectin, µg/g; *n* = 80	886.4 ± 1123.2
CRP, mg/L; *n* = 40	33.4 ± 65.3

All data are mean ± SD unless otherwise indicated

*BMI* body mass index, *CRP* C-reactive protein

Approximately 50% of all participants had pancolitis; 25% were diagnosed with left-sided colitis and 25% with proctosigmoiditis (Table 2). Regarding the disease history at diagnosis, extraintestinal manifestations were reported for roughly 7% of all participants, of which the most common were arthralgia/arthritis (5.9%). About one fifth of all patients had a documented history of anaemia (*n* = 58), and weight loss was reported for 93 (30.3%) participants. No history of tobacco use was reported for 184 (59.9%) patients, with 46 (15.0%) and 76 (24.8%) being current and former tobacco users, respectively.

**Table 2:** Disease location and selected parameters

Parameter	*n* (%)
Disease location at diagnosis	
Proctosigmoiditis	76 (24.8)
Left-sided colitis	76 (24.8)
Extensive colitis	154 (50.2)
N/A	1 (0.3)
History of extraintestinal manifestations at diagnosis	
Arthralgia/arthritis	18 (5.9)
Uveitis	3 (1.0)
Erythema nodosum	1 (0.3)
Anaemia at baseline	
Yes	58 (18.9)
No	190 (61.9)
N/A	59 (19.2)
Weight reduction at diagnosis	
Yes	93 (30.3)
No	209 (68.1)
N/A	5 (1.6)
Tobacco use at baseline	
No	184 (59.9)
Yes	46 (15.0)
Former	76 (24.8)
N/A	1 (0.3)

*N/A*, not available

Figure 1 depicts the patient disposition and the proportion of patients with need for IS and/or anti–TNF-α therapy and/or hospitalisation. Of the 307 patients in the analysis population, 209 (68.1%) were neither hospitalised nor received immunosuppressive therapy and were thus classified as having an uncomplicated disease course (group 1). In contrast, 98 (31.9%) patients were classified as having a complicated disease course, requiring hospitalisation and/or IS and/or anti–TNF-α therapy (group 2). Within group 2, 56 (57.1%) were treated with anti–TNF-α therapy either with or without IS, and 42/98 (42.9%) received immunosuppressive monotherapy with azathioprine.

When assessing prior medication, we found that 264 (86.0%) patients had been exposed to non-immunosuppressive therapy, the most common of which was 5-ASA (Table 3). Topical 5-ASA use was reported by 110 (41.7%) patients, and 226 (85.6%) participants reported receiving oral 5-ASA formulations. Prior use of oral budesonide and systemic corticosteroids was documented for 49 (18.6%) and 108 (40.9%) patients, respectively.

**Table 3:** Non-immunosuppressive medication at baseline

Parameter	Non-immunosuppressive medication, *n* (%) (*n* = 264)	
	**Yes**	**No**
5-ASA		
Topic	110 (41.7)	154 (58.3)
Oral	226 (85.6)	38 (14.4)
Budesonide, oral	49 (18.6)	215 (81.4)
Systemic corticosteroids	108 (40.9)	156 (59.1)

*5-ASA*, 5-aminosalicylic acid

At baseline, the mean ± SD disease activity score for the entire cohort as assessed through SCCAI was 3.83 ± 3.16 (Table 4). At baseline, 195 patients (63.5%) were in clinical remission (defined as SCCAI score < 5). The average SCCAI score decreased during the 24-month study period, reaching 1.86 ± 2.4 after 12 months and 1.30 ± 2.10 after 24 months. At the end of the study period, all but 11 patients (*n* = 190) were in clinical remission.

**Table 4:** Potential prognostic factors evaluated at diagnosis or baseline visit

Parameter	Analysis population		
	**Total patients (*n* = 307)**	**Uncomplicated disease course (*n* = 209)**	**Complicated disease course (*n* = 98)**
Age (years, mean ± SD)	38.5 ± 15.6	39.8 ± 15.8	35.7 ± 14.7
Sex			
Male	170 (55.4)	110 (52.6)	60 (61.2)
Female	137 (44.6)	99 (47.4)	38 (38.8)
SCCAI (mean ± SD) severity, SCCAI	3.83 ± 3.16	3.78 ± 3.12	3.95 ± 3.26
Use of corticosteroids			
Yes	120 (39.1)	65 (31.1)	55 (56.1)
No	187 (60.9)	144 (68.9)	43 (43.9)
Clinical response to corticosteroids			
Yes	30 (9.8)	18 (8.6)	12 (12.2)
No	277 (90.2)	191 (91.4)	86 (87.8)
Anaemia			
Yes	88 (28.7)	48 (23.0)	40 (40.8)
No	219 (71.3)	161 (77.0)	58 (59.2)
EIM			
Yes	25 (8.1)	13 (6.2)	12 (12.2)
No	282 (91.9)	196 (93.8)	86 (87.8)
Smoking			
Yes	48 (15.6)	37 (17.7)	11 (11.2)
No	259 (84.4)	172 (82.3)	87 (88.8)
Former smoker			
Yes	76 (24.8)	55 (26.3)	21 (21.4)
No	231 (75.2)	154 (73.7)	77 (78.6)
Mayo endoscopic subscore, 3			
Yes	55 (17.9)	28 (13.4)	27 (27.6)
No	252 (82.1)	181 (86.6)	71 (72.4)
Thrombocytosis			
Yes	43 (14.0)	28 (13.4)	15 (15.3)
No	264 (86.0)	181 (86.6)	83 (84.7)

All data are *n* (%) unless otherwise stated

*EIM* extraintestinal manifestations, *SCCAI* simple clinical colitis activity index

Of the 98 patients requiring immunosuppressive therapy, 56 (57.1%) were treated with TNF antibodies (adalimumab, golimumab, infliximab); 42 received monotherapy with azathioprine. The median time from baseline until initiation of immunosuppressive therapy was 5.3 months. Of all patients, 23.1% (*n* = 71) required at least one unscheduled visit to a physician, with one patient needing 13 unscheduled visits.

### Predictive parameters for a complicated disease course

Examining potential differences of baseline characteristics, we found that patients with a complicated disease course were younger (mean age, 35.7 vs 39.8 years, *p* = 0.02 Mann–Whitney *U* test), and more frequently reported weight reduction (45.9% vs 23.0%, *p* < 0.001, chi-square test) at the time of diagnosis than patients with an uncomplicated disease course. Moreover, they had higher rate of severe Mayo endoscopic scores at baseline (*p* = 0.033; Supplementary Table S1). We performed a logistic regression analysis to identify baseline parameters that are predictive of a complicated disease course. A list of all examined prognostic factors is provided in Table 4. For this analysis, we relied on the measurements performed at the baseline visit, except for corticosteroid use, which here refers to not only corticosteroid use at baseline but also to previous UC-related corticosteroid use by the patient.

In a first step, all demographic and anamnestic parameters observed in ≥ 5% of patients were analysed with regard to a potential predictive value in both uni- and multivariate regression models (Table 5). All parameters with *p* < 0.05 in either the uni- or multivariate model (age, male sex, use of corticosteroids, anaemia, and severe Mayo endoscopic subscore of 3 points) were then included in a second multivariate model (Table 6). Parameters that were significant at *p* < 0.05 in this reduced model were then stepwise selected to be included in an optimised mode. In this final model, prior use of corticosteroids and anaemia were associated with a 2.3- and 1.9-fold increased risk for subsequent need for immunosuppressive therapy and/or hospitalisation, respectively.

**Table 5:** Uni- and multivariate Cox regression analysis for predictive factors of complicated disease course (*n* = 307)

Factor, risk	Univariate analysis		Multivariate analysis, complete model	
	**Hazard ratio (95% CI)**	***p* value**	**Hazard ratio (95% CI)**	***p* value**
Age, years	0.983 (0.969–0.997)	**0.014**	0.985 (0.971–1.000)	**0.047**
Sex, male	1.517 (1.007–2.286)	**0.044**	1.490 (0.980–2.267)	0.062
Disease severity, SCCAI	1.035 (0.974–1.099)	0.273	1.038 (0.977–1.103)	0.227
Corticosteroids, yes	2.540 (1.700–3.795)	** < 0.001**	2.085 (1.318–3.299)	**0.002**
Clinical response to corticosteroids, no	1.505 (0.822–2.755)	0.208	1.074 (0.555–2.077)	0.832
Anaemia, yes	2.200 (1.467–3.298)	** < 0.001**	1.995 (1.265–3.145)	**0.003**
EIM, yes	1.699 (0.928–3.112)	0.108	1.661 (0.883–3.124)	0.115
Smoking, yes	0.638 (0.341–1.196)	0.137	0.601 (0.316–1.140)	0.119
Former smoker, yes	0.858 (0.529–1.391)	0.535	1.068 (0.637–1.790)	0.803
Mayo endoscopic subscore = 3, yes	1.863 (1.188–2.921)	**0.007**	1.251 (0.776–2.015)	0.358
Thrombocytosis, yes	1.262 (0.728–2.189)	0.407	0.712 (0.380–1.334)	0.288

*EIM* extraintestinal manifestations, *SCCAI* simple clinical colitis activity index

**Table 6:** Reduced and optimised Cox regression analysis for predictive factors of complicated disease course (*n* = 307)

Factor, risk	Multivariate analysis			
	**Reduced model**		**Optimised model**	
	**Hazard ratio (95%CI)**	***p* value**	**Hazard ratio (95%CI)**	***p* value**
Age, years	0.987 (0.973–1.001)	0.079	–	–
Sex, male	1.448 (0.959–2.188)	0.078	–	–
Corticosteroids, yes	2.027 (1.321–3.111)	**0.001**	2.326 (1.550 – 3.491)	** < 0.001**
Anaemia, yes	1.784 (1.171–2.718)	**0.007**	1.940 (1.288 – 2.922)	**0.002**
Mayo endoscopic subscore = 3, yes	1.361 (0.851–2.178)	0.198	–	–

Based on the two parameters (therapy with systemic corticosteroids and anaemia), a predictive risk model was developed to determine the individual patient’s likelihood of experiencing a complicated disease course at 6, 12, or 24 months (Table 7).

**Table 7:** Risk model for prediction of complicated disease course

Time	Corticosteroids	Anaemia	Probability of IS, %
After 6 months	No	No	10.5
	Yes	No	15.1
	No	Yes	13.7
	Yes	Yes	19.6
After 12 months	No	No	17.0
	Yes	No	24.0
	No	Yes	21.9
	Yes	Yes	30.7
After 24 months	No	No	23.0
	Yes	No	32.1
	No	Yes	29.5
	Yes	Yes	40.3

## Discussion

EPICOL was a prospective study on a large inception cohort of patients with UC to identify early predictors for a complicated disease course.

We found that approximately one third (98/311 [31.6%]) of all patients in our study received IS and/or biologic treatment. Only one patient was admitted to the hospital; this admission occurred after the 24-month follow-up period. A possible explanation for the low number of hospitalisations is that our study was conducted exclusively at specialised IBD centres and, therefore, tighter disease management may have been observed, resulting in a lower hospitalisation rate. This may also be reflected in the high number of unscheduled visits to physicians, which enabled immediate therapeutic changes where necessary, thereby potentially avoiding hospitalisations. This notion is supported by the fact that the mean SCCAI score of our cohort declined continuously over the course of this study, reaching a value of 1.3 ± 2.1 at the end of the follow-up period. It is also worth mentioning that other studies have found that EIMs may be present in up to 25% of patients with IBD before diagnosis [25], which is significantly higher than the ~ 7% of patients in our cohort who reported EIMs in their disease history. We speculate that this lower rate of EIMs reported before diagnosis may, at least in part, be caused by the fact that EIMs were not the primary methodologic concern of this study, leading to some degree of underreporting compared with other studies that focused particularly on EIMs.

The most important finding of our study is that prior use of corticosteroids or anaemia at diagnosis were associated with a significantly higher risk for a complicated disease course, defined as need for treatment with IS and/or biologics and/or UC-related hospitalisation. Although numerous studies have already found an association between prior corticosteroid use and poorer outcomes, most were either retrospective [17, 18, 26–28]; addressed other outcomes, such as risk for colectomy or proximal disease extension [18, 26–29]; or could not validate prior corticosteroid use as an independent predictor in rigorous multivariate analysis [26]. To the best of our knowledge, this is, therefore, the first prospective study that demonstrates early use of corticosteroids as an independent predictor of requiring treatment with IS and/or biologics and/or hospitalisation.

Based on our findings, we developed a risk model for the prediction of a complicated disease course in 6, 12, and 24 months after baseline for recently diagnosed patients with UC. In this model, the previous use of corticosteroids and/or anaemia increases the risk of immunosuppressive therapy after 6 months to > 13%. The likelihood of a complicated disease course occurring after 12 and 24 months is quite high, ranging from 21.9% (after 12 months with anaemia) to 40.3% (after 24 months with previous corticosteroid use and anaemia). With regard to the risk model that we developed for patients with CD, it is interesting that an age at onset of < 40 years was identified as risk factor as well as anaemia and corticosteroid use. However, the rather low number of patients with UC with a complicated disease course in our cohort is in line with published results demonstrating a less complicated disease course for patients with UC in a certain time frame compared with patients with CD [30]. Both models are suitable for identifying patients at risk for a more complex disease course. Certainly, further verification of our models incorporating independent patient cohorts is required in future.

Multiple studies have already revealed an association between anaemia and unfavourable disease events, such as corticosteroid utilisation [31], corticosteroid-refractory disease [32], colectomy [33, 34], and relapse following aminosalicylate treatment [35]. As in the case of corticosteroid use, these were, however, mostly retrospective analyses. Our study thus provides validation of the predictive power of anaemia within a prospective setting. Interestingly, anaemia was also an independent predictor of a complicated disease course in our EPIC study on patients with recently diagnosed CD [24]. The cause of anaemia in IBD is multifactorial, with important factors being blood loss through active UC, impaired iron absorption, and utilisation caused by chronic inflammation as well as reduced intake and, less frequently, vitamin B12 deficiency [36]. Because we did not observe a baseline difference in the SCCAI parameter “blood in stool” between our two groups (data not shown), it could be speculated that, in our cohort, an anaemic state at baseline or diagnosis reflects reduced iron absorption rather than secondary blood loss, which could in turn explain the predictive power of this parameter.

However, given the general scarcity and limited suitability of prospective studies describing clinical predictors for recently diagnosed patients, it seems worth posing the question as to how far the disease course can be accurately predicted solely based on clinical parameters. Future studies will need to address this issue and investigate whether genomic, transcriptomic, and microbiomic profiling approaches can achieve a greater predictive power than clinical parameters alone.

The main strengths of our study are its prospective and multi-centre design, the high number of patients and study centres, as well as the short time interval between diagnosis and enrolment. Nonetheless, there are several limitations. One limitation is that the investigations were conducted exclusively at IBD-specialised centres, meaning that patients treated by primary care physicians were not included in this study. As a result, we cannot exclude the possibility that different or additional predictors may be relevant in patients treated at centres with less therapeutic experience. In addition, a substantial fraction of all measurements were not available in some cases, limiting our analysis to sufficiently well-reported parameters.

In summary, this thoroughly designed, large, prospective inception cohort study of patients with UC determined clinical predictors that are easy to assess in everyday clinical practice and provided important evidence for the risk stratification of patients with recently diagnosed UC.

## Supplementary information

Below is the link to the electronic supplementary material.

Supplementary file1 (DOCX 15 KB)

## Data Availability

All data and materials support the claims in this manuscript and comply with field standards.
