# Supplementary material for: Clinical predictors for a complicated course of disease in an inception cohort of patients with ulcerative colitis: results from the prospective, observational EPICOL study
Source: Int J Colorectal Dis. 2022 Jan 27;37(2):485–93. doi: 10.1007/s00384-022-04098-7 (PMC8803753; doi:10.1007/s00384-022-04098-7)
Supplement: Supplementary file 1 — Supplementary file1 (DOCX 15 KB) [file 384_2022_4098_MOESM1_ESM.docx]

Supplementary Information

Members of the EPICOL Study Group

Dr. med. Repp, Dr. med. Petra, Dr. med. Heil, Dr. med. Hahn, Dr. med. Brüning, Dr.med. Lange, Dr.med. Söllenböhmer, Dipl. med. Schache, Dr. med. Liceni, Dr. med. Spitz, PD Dr. med. Büning, Dr. med. Aminalai, Dr. med. Becker, Dr. med. Grüngreiff, Dmed. Palmai, Dr. med. Behrendt, Dr. med. Leo, Dr. med. Könemann, Dr. med. Geißler, Dr. med. Witzemann, Dr. med. Wübbolding, Dr. med. Simonis, Dr. med. Franke, Dr. med. Frach, Dr. med. Weisflog, Prof. Dr. med. Janisch, Dr. med. Pelster, Dr. med. Kihn, Prof. Dr. med.Dignass, Dr.med. Günther, Dr. med. Schmitz, Dr. med. Naumann, Dr. med. Krummenerl, Dr. med. Zeisler, Prof. Dr. med. Kühbacher, Dr. med. Halle, Dr. med. Miks, Dr. med. Müller-Ziehm, Dr. med. Felten, Dr. med. Engelke, Dr. med. Muhl-Gozdowsky, Dr.med. Schwarz, Prof. Dr. med. Stallmach, Dr. med. Schulze, Dr. med. Schulte Bockholt, Prof. Dr. med. Langmann, Dr. med. Torff, Dr. med. Benner, Dr. med. Dierkes-Globisch, Prof. Dr. med. Kruis, Dr. med Schneider, Dr. med. Schwerdtfeger, Dr. med. Teich, Prof. Dr. med. Schiefke, Prof. Dr. med. Fellermann, Dr. med. Kilian, Dr. med. Mackenroth, Dr. med. Gratz, Dr. med. Bokemeyer, Dr. med. Hundegger, Dr. med. Schweitzer, Dr. med. Linnepe, PD Dr. Pace, Dr. med. Hoesl, Dr. med. Behrends, Dr.med. Emke, Dr. med. Grümmer, Dr. med. Brandt, Dr. med. Hopert, Dr. med. Ringel, Mrs. Kleinfeldt, Dr. med. Bank, Prof. Dr. med. Zahn, Prof. Dr. med. von Boyen, Dr. med. Rehbehn, Dr. med. Kinzel, Dr. med. Schumann, Dr. med. Krichbaum, Mr. Wölkner, Prof. Dr. med. Löhr, Dr. med. Schirin-Sokhan, Dr. med. Dreier, Dr. med. Hoffstadt, Dr. med. Frölich.

Table S1 Mayo endoscopic subscore for patients with UC at baseline^a^

|  |  | **Uncomplicated** | **Complicated** |
| --- | --- | --- | --- |
| **Baseline Mayo**  **endoscopic score, n (%)** | **Total patients**  **(n = 307)** | **disease course**  **(n = 209)** | **disease course**  **(n = 98)** |
| Normal | 2 (0.6) | 2 (1.0) | 0 (0.0) |
| Mild | 88 (28.7) | 62 (29.7) | 26 (26.5) |
| Moderate | 159 (51.8) | 115 (55.0) | 44 (44.9) |
| Severe | 55 (17.9) | 28 (13.4) | 27 (27.6) |
| N/A | 3 (1.0) | 2 (1.0) | 1 (1.0) |

^a^p = 0.033, Fisher exact test

*NA* not available, *UC* ulcerative colitis
